# Evaluation and Comparison of the Predictive Value of 4C Mortality Score, NEWS, and CURB-65 in Poor Outcomes in COVID-19 Patients: A Retrospective Study from a Single Center in Romania

**DOI:** 10.3390/diagnostics12030703

**Published:** 2022-03-13

**Authors:** Cosmin Citu, Florin Gorun, Andrei Motoc, Adrian Ratiu, Oana Maria Gorun, Bogdan Burlea, Octavian Neagoe, Ioana Mihaela Citu, Ovidiu Rosca, Felix Bratosin, Mirela Loredana Grigoras, Raul Patrascu, Daniel Malita

**Affiliations:** 1 Department of Obstetrics and Gynecology, “Victor Babes” University of Medicine and Pharmacy Timisoara, 2 Eftimie Murgu Square, 300041 Timisoara, Romania; citu.ioan@umft.ro (C.C.); ratiu.adrian@umft.ro (A.R.); 2 Department of Anatomy and Embryology, “Victor Babes” University of Medicine and Pharmacy Timisoara, 2 Eftimie Murgu Square, 300041 Timisoara, Romania; amotoc@umft.ro (A.M.); grigoras.mirela@umft.ro (M.L.G.); 3 Department of Obstetrics and Gynecology, Municipal Emergency Clinical Hospital Timisoara, 1–3 Alexandru Odobescu Street, 300202 Timisoara, Romania; oanabalan@hotmail.com (O.M.G.); bogdanburlea@yahoo.com (B.B.); 4 First Department of Surgery, Second Discipline of Surgical Semiology, “Victor Babes” University of Medicine and Pharmacy, Eftimie Murgu Sq. Nr. 2, 300041 Timisoara, Romania; dr.octavian.neagoe@gmail.com; 5 Department of Internal Medicine I, “Victor Babes” University of Medicine and Pharmacy Timisoara, 2 Eftimie Murgu Square, 300041 Timisoara, Romania; citu.ioana@umft.ro; 6 Methodological and Infectious Diseases Research Center, Department of Infectious Diseases, “Victor Babes” University of Medicine and Pharmacy, 300041 Timisoara, Romania; ovidiu.rosca@umft.ro (O.R.); felix.bratosin7@gmail.com (F.B.); 7 Department of Functional Sciences, “Victor Babes” University of Medicine and Pharmacy Timisoara, Eftimie Murgu Square 2, 300041 Timisoara, Romania; patrascu.raul@umft.ro; 8 Department of Radiology, “Victor Babes” University of Medicine and Pharmacy Timisoara, Eftimie Murgu Square nr. 2, 300041 Timisoara, Romania; malita.daniel@umft.ro

**Keywords:** COVID-19, prediction, mortality, 4C Mortality, NEWS, CURB-65

## Abstract

To date, the COVID-19 pandemic has caused millions of deaths across the world. Prognostic scores can improve the clinical management of COVID-19 diagnosis and treatment. The objective of this study was to assess the predictive role of 4C Mortality, CURB-65, and NEWS in COVID-19 mortality among the Romanian population. A single-center, retrospective, observational study was conducted on patients with reverse transcriptase-polymerase chain reaction (RT-PCR)-proven COVID-19 admitted to the Municipal Emergency Clinical Hospital of Timisoara, Romania, between 1 October 2020 and 15 March 2021. Receiver operating characteristic (ROC) and area under the curve (AUC) analyses were performed to determine the discrimination accuracy of the three scores. The mean values of the risk scores were higher in the non-survivors group (survivors group vs. non-survivors group: 8 vs. 15 (4C Mortality Score); 3 vs. 8.5 (NEWS); 1 vs. 3 (CURB-65)). In terms of mortality risk prediction, the NEWS performed best, with an AUC of 0.86, and the CURB-65 score performed poorly, with an AUC of 0.80. CURB-65, NEWS, and 4C Mortality scores were significant mortality predictors in the analysis, with acceptable calibration. Among the scores assessed in our study, NEWS had the highest performance in predicting in-hospital mortality in COVID-19 patients. Thus, the findings from this study suggest that the use of NEWS may be beneficial to the early identification of high-risk COVID-19 patients and the provision of more aggressive care to reduce mortality associated with COVID-19.

## 1. Introduction

In March 2020, WHO declared the new coronavirus outbreak (COVID-19) a global pandemic due to the rapidly increasing number of SARS-CoV-2 infections reported since December 2019 [1]. By 13 February 2022, 410,848,671 cases of COVID-19 and 5,829,566 deaths had been reported worldwide. During this time, 2,531,597 cases of COVID-19 and 61,376 deaths had been registered in Romania [2]. Patients affected by COVID-19 may be asymptomatic; may experience mild symptoms; or may develop atypical pneumonia, respiratory failure and acute respiratory distress syndrome (ARDS) (Appendix A–Table A1), which may lead to death [3,4,5]. Prognostic scores can improve the clinical management of COVID-19 diagnosis and treatment. Early identification of severe cases can lead to more aggressive care and lower mortality. Studies investigating the accuracy of these scores can provide important data for large-scale clinical implementation. Several prognostic scores have been developed to identify the high risk of death in patients with poor outcomes [6,7]. The 4C Mortality Score (where 4C refers to Coronavirus Clinical Characterization Consortium) is a validated predictor of mortality in hospitalized patients with COVID-19 [8,9]. CURB-65 is a commonly applied severity score in community-acquired pneumonia (CAP) management [10,11]. The National Early Warning Score (NEWS) was developed in 2012 in the United Kingdom by the NEWS Development and Implementation Group on behalf of the Royal College of Physicians [12,13]. The objective of the present study was to assess the predictive ability of 4C Mortality, CURB-65, and NEWS in COVID-19 mortality among the Romanian population.

## 2. Materials and Methods

### 2.1. Study Design and Setting

A single-center, retrospective, observational study was conducted on patients confirmed to be COVID-19 positive based on the reverse transcription-polymerase chain reaction (RT-PCR) test, and admitted to the Municipal Emergency Clinical Hospital of Timisoara, Romania, between 1 October 2020 and 15 March 2021. The study was approved by the Ethics Committee of the University of Medicine and Pharmacy “Victor Babes” Timisoara (No. 22726/17 November 2021).

### 2.2. Participants

Participants enrolled in the study met the following criteria: (1) admitted to the Municipal Emergency Clinical Hospital following a positive test for SARS-CoV-2 by real-time reverse transcriptase chain reaction (RT-PCR) on a nasopharyngeal swab between 1 October 2020 and 15 March 2021; (2) had complete clinical and laboratory data documented in electronic medical records; (3) aged over 18 years. Patients under 18 years of age or with missing data were excluded.

### 2.3. Data Sources Measurement and Outcome

Demographic, clinical, and outcome data were collected from electronic medical records using a prespecified case report form. Demographic data included age and gender. Clinical data included comorbidities and presenting symptoms or signs. Clinical outcomes were also examined, including admission to the intensive care unit, mechanical ventilation, and mortality. The 4C Mortality Score incorporates eight independent parameters: age, gender, number of comorbidities, respiratory rate, peripheral oxygen saturation level, Glasgow Coma Scale, blood urea nitrogen level, and C-reactive protein level. CURB-65 involves five easily obtainable parameters: new-onset confusion, urea >7 mmol/L, respiratory rate ≥30/minute, systolic blood pressure <90 mmHg and/or diastolic blood pressure ≤60 mmHg, and age ≥65 years. Each parameter present is scored with 1 point. The National Early Warning Score is based on six parameters that can be obtained from hospitalized patients or those in the emergency department: respiratory rate, oxygen saturation, temperature, systolic blood pressure, pulse rate, and level of consciousness. The clinical endpoint was in-hospital mortality.

### 2.4. Statistical Analysis

Statistical analysis was performed using RStudio. Categorical variables were reported as absolute number (n) and relative frequency (%), and were compared using Fisher’s exact test. Depending on the normality of the distribution, continuous variables were represented as a median (interquartile range) or as a mean (±SD). The Mann–Whitney test was used to compare non-parametric data. The Shapiro–Wilk test was used to assess the normality of the distribution. The receiver operating characteristic area under the curve was examined to identify the accuracy of discrimination of the evaluated scores. Binary logistic regression was used to determine the independent predictive value of all the risk scores.

## 3. Results

### 3.1. Baseline Characteristics

Our study included 133 COVID-19 patients confirmed by RT-PCR, of which 18 (13.5%) died (Table 1). The median age was 65 years (IQI = 21), with more males (51.1%) than females. The most common comorbidities were hypertension (65.4%) and chronic kidney disease (51.9%) (Table 1).

### 3.2. Comparison of 4C Mortality Score, NEWS and CURB-65 Scores

The mean values of the risk scores were higher in the non-survivors group: 4C Mortality Score, 8 (IQI = 8) vs. 15 (IQI = 5.35); NEWS, 3 (IQI = 4) vs. 8.5 (IQI =4.5); and CURB-65, 1 (IQI = 2) vs. 3 (IQI = 1.75). Mortality rates within the 4C Mortality Score risk groups were 0% (Low), 5.56% (Intermediate), 38.9% (High), and 55.6% (Very High) (Table 2).

**Figure 1:**
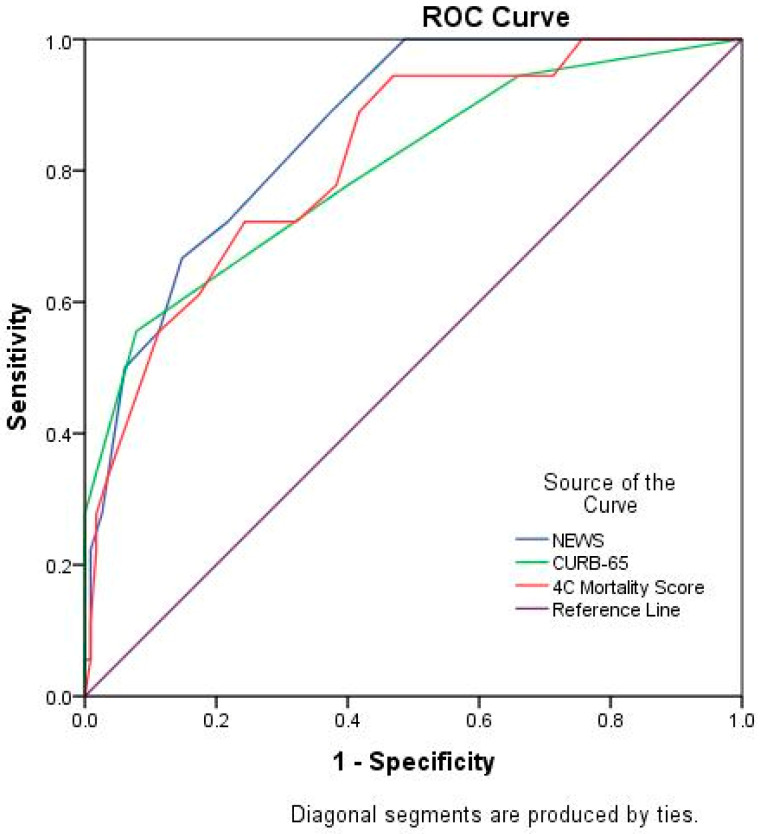
Receiver operating characteristic curve of clinical risk scores in predicting mortality.

To determine the discrimination accuracy of the 4C Mortality Score, NEWS, and CURB-65 score, a ROC-AUC analysis was performed (Figure 1). The most accurate scale was the NEWS, with an AUC value of 0.861 (95% CI 0.784–0.939) (Table 3).

In terms of mortality risk prediction, the NEWS performed best, with an AUC of 0.86. The CURB-65 score performed poorly, with an AUC of 0.80 (Table 3).

Table 4 shows the mortality accuracy measures for cut-offs 3, 8, 11, and 14 at the 4C Mortality Score. The sensitivity of each cut-off ranged from 100% for 4C > 3 and NEWS > 3 to 55% for 4C > 14. Specificity ranged from 24% for NEWS > 3 to 92% for CURB-65 > 2.

### 3.3. Association of 4C Mortality Score, NEWS, and CURB-65 Scores with COVID-19 Mortality

**Figure 2:**
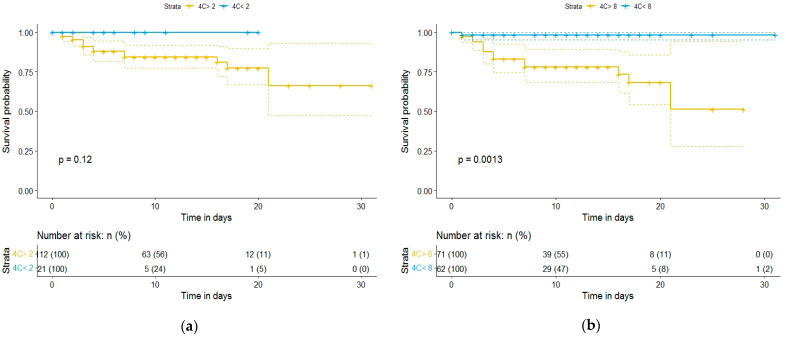
Kaplan–Meier survival curves of hospitalized COVID-19 patients: (**a**) according to 4C Mortality Score cutoff value of 2; (**b**) according to 4C Mortality Score cutoff value of 8.

**Figure 3:**
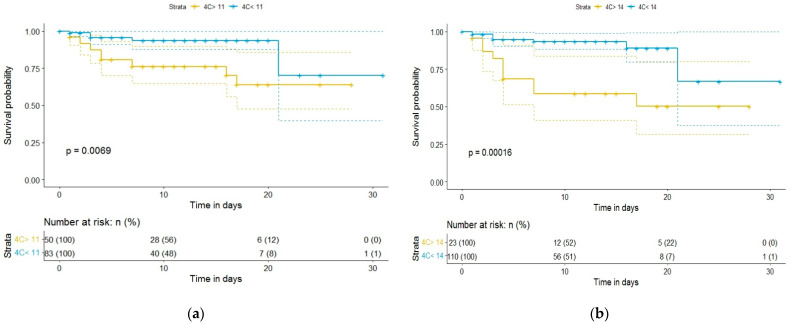
Kaplan–Meier survival curves of hospitalized COVID-19 patients: (**a**) according to 4C Mortality Score cutoff value of 11; (**b**) according to 4C Mortality Score cutoff value of 14.

Kaplan–Meier curves and a univariate binary regression model were created using various cutoff points for 4C Mortality Score, NEWS, and CURB-65. Figure 2 and Figure 3 show the Kaplan–Meier survival curve as a function of the 4C Mortality Score cutoff values. Differences in survival for patients above the baseline 4C Mortality Score of 8, 11, and 14, compared to patients with scores below these values, were statistically significant (Figure 2 and Figure 3). However, differences in survival for patients with 4C Mortality Score values above the threshold of 2, compared to those below the threshold, were not statistically significant (*p* = 0.12).

**Figure 4:**
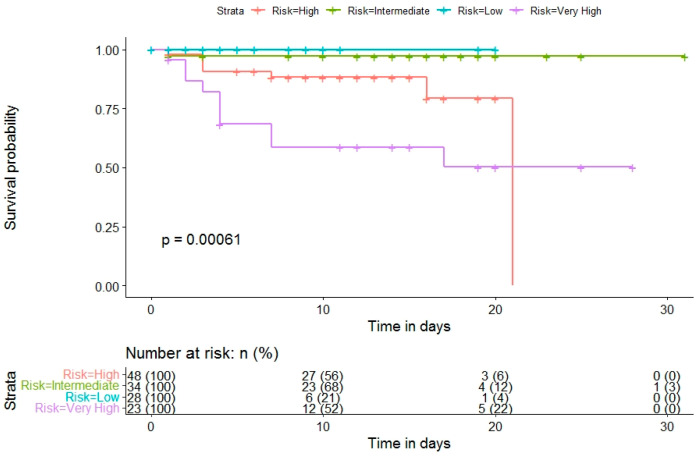
Kaplan–Meier survival curves of hospitalized COVID-19 patients according to 4C Mortality Score risk.

In addition, differences in patient survival by 4C Mortality Score classification showed highly statistically significant differences (*p* < 0.001) (Figure 4).

**Figure 5:**
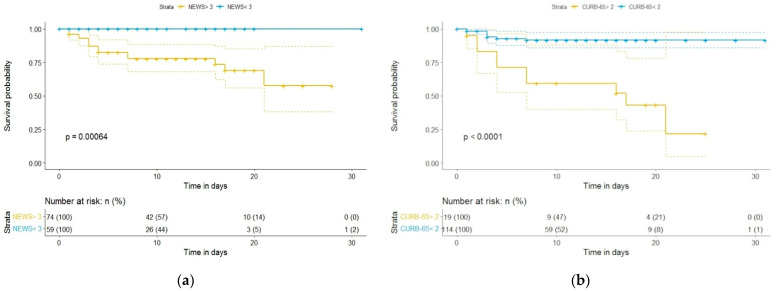
Kaplan–Meier survival curves of hospitalized COVID-19 patients: (**a**) according to NEWS cutoff value of 3; (**b**) according to CURB-65 cutoff value of 2.

Differences in survival for patients with NEWS and CURB-65 scores above the baseline, compared to those below the baseline, were highly statistically significant (*p* < 0.001 for each) (Figure 5).

Univariate regression analysis was performed to determine the link between the risk scores and in-hospital mortality. All risk scores were significant mortality predictors in the analysis, with acceptable calibration. The results are presented in Table 5.

## 4. Discussion

The predictive role of 4C Mortality Score, CURB-65, and NEWS in COVID-19 mortality has not been previously evaluated in the Romanian population. Thus, we evaluated these scores among 133 patients confirmed to be COVID-19 positive by RT-PCR who were admitted to the Municipal Emergency Clinical Hospital of Timisoara, Romania. The mortality rate among these patients was 13.5%, which is lower than the mortality rate reported in a previous article conducted at the Municipal Emergency Clinical Hospital of Timisoara in another study period. The mortality rate is also lower than the 17% mortality rate found in a systematic review among inpatients [14,15]. Age, cardiovascular disease, and chronic kidney disease were associated with increased mortality among patients in this study.

In this study, the 4C Mortality Score provided acceptable specificity and sensitivity in predicting mortality in all patients. We observed an AUC of 0.81, which is higher than the deviation in the original research of 0.79 [8]. In addition, the mortality rates of the Low, Intermediate, High, and Very High risk groups on the 4C Mortality Score were 0.0%, 5.56%, 38.9%, and 55.6%, respectively. These are similar to those reported in the original study, which reported rates of 1.2%, 9.9%, 31.4%, and 61.5%, respectively.

The CURB-65 score, which consists of five parameters (confusion, blood urea nitrogen, respiratory rate, blood pressure, and age), was implemented in 2003 by Lim et al. to easily predict the increased risk of 30-day mortality in patients with CAP [13]. A study conducted by Guo et al. in a Wuhan hospital showed that CURB-65 had good performance in determining mortality, with an AUC of 0.81 [10]. In addition, a study of 481 patients with COVID-19 conducted by Doganay and Rohat demonstrated a good performance of CURB-65 (AUC 0.84) in predicting in-hospital mortality [16]. Consistent with these studies, in our study CURB-65 had a good performance, with an AUC of 0.801, in predicting mortality in COVID-19 patients.

The NEWS is a score implemented by the NEWS Development and Implementation Group on behalf of the Royal College of Physicians in 2012, and uses six parameters for the assessment and response to acute illness: respiratory rate, oxygen saturation, temperature, systolic blood pressure, pulse rate, and level of consciousness [12]. Kostakis et al. demonstrated in a study that NEWS had good performance in predicting mortality among COVID-19 patients [17]. Pokeerbux et al., in a study conducted on a French cohort that included hospitalized COVID-19 patients, showed that NEWS was an independent predictor of ICU transfer and in-hospital mortality, with an AUC of 0.82 [18]. Our study also found that NEWS had higher performance (AUC of 0.861) in predicting mortality among COVID-19 patients, and was the most accurate scale. However, Lim et al. suggest that NEWS be adjusted with a more sensitive score for oxygen demand in order to more accurately consider the development of hypoxic respiratory failure in COVID-19 patients [19].

In addition to these scores, another method of increasing the speed of combating COVID-19 is artificial intelligence. Artificial intelligence applications in clinical settings play an important role in improving the productivity and efficiency of studies involving large samples, with the intention of higher degrees of accuracy in prediction. Another benefit of artificial intelligence is that it can speed up the drug development process in emergency situations, as seen in the COVID-19 pandemic [20,21].

Therefore, prediction scores coupled with artificial intelligence can be particularly important for decision-making needs in crisis situations such as the COVID-19 pandemic.

This study has some limitations. First, the study follows a retrospective design and is based on data from a single health clinic. Second, the sample may not have been large enough to assess the predictive performance of 4C Mortality Score, NEWS, and CURB-65 for death, as there were only 18 deaths in this cohort. In addition, the baseline demographics of survivors and non-survivors were very different; there was a higher percentage of comorbidities among non-survivors.

## 5. Conclusions

All of the risk scores evaluated were significant mortality predictors in the analysis, with acceptable calibration. Among the scores assessed in our study, NEWS had a higher performance in predicting in-hospital mortality in COVID-19 patients. The findings from this study suggest that the NEWS may be beneficial in the early identification of high-risk COVID-19 patients and in providing more aggressive care to reduce mortality associated with COVID-19.

## Figures and Tables

**Table 1:** Baseline characteristics of 133 COVID-19 patients.

Variables	Overall (n = 133)	Survivors (n = 115)	Died (n = 18)	*p*-Value
***Demographics***				
Gender (n/%)				
Female	65/48.9%	58/50.4%	7/38.9%	0.45 (OR = 1.59)
Male	68/51.1%	57/ 49.6%	11/61.1%	
Age (years; median (IQR))	65 (21)	62 (20.5)	70 (15.5)	0.02
***Comorbidities*** (n/%)				
Hypertension	87/65.4%	72/62.6%	15/83.3%	0.11 (OR = 2.96)
Diabetes	59/44.4%	49/42.6%	10/55.6%	0.32 (OR = 1.67)
Cardiovascular disease	44/33.1%	32/27.8%	12/66.7%	0.002 (OR = 5.11)
CKD	69/51.9%	55/47.8%	14/77.8%	0.02 (OR = 3.78)
COPD/asthma	26/19.5%	20/17.4%	6/33.3%	0.12 (OR = 2.35)
Cancer	15/11.3%	11/9.6%	4/22.2%	0.12 (OR = 2.67)
***Presenting symptoms*** (n/%)				
Cough	77/57.9%	65/56.5%	12/66.7%	0.45 (OR = 1.53)
Dyspnea	69/51.9%	59/51.3%	10/55.6%	0.80 (OR = 1.18)
Chest pain	18/13.5%	14/12.2%	4/22.2%	0.26 (OR = 2.04)
Fatigue	82/61.7%	68/59.1%	14/77.8%	0.19 (OR = 2.40)
Myalgia	33/24.8%	28/24.3%	5/27.8%	0.77 (OR = 1.19)
No smell/taste	18/13.5%	17/14.8%	1/5.56%	0.46 (OR = 0.34)
Gastrointestinal symptoms	52/39.1%	44/38.3%	8/44.4%	0.61 (OR = 1.28)
***Clinical course*** (n/%)				
Mechanic ventilation	9/6.77%	2/1.74%	7/38.9%	<0.001 (OR = 33.8)
ICU admission	10/7.52%	4/3.48%	6/33.3%	<0.001 (OR = 13.3)

COPD, chronic obstructive pulmonary disease; CKD, chronic kidney disease; ICU, intensive care unit.

**Table 2:** Comparison of 4C Mortality, NEWS, and CURB-65 scores.

Score	Overall	Survived	Died	*p*-Value
4C Mortality Score median (IQI)	9 (9)	8 (8)	15 (5.25)	<0.001
Low (0–3) n (%)	28 (21.1%)	28 (24.3%)	-	
Intermediate (4–8) n (%)	34 (25.6%)	33 (28.7%)	1 (5.56%)	
High (9–14) n (%)	48 (36.1%)	41 (35.7%)	7 (38.9%)	
Very high (15–21) n (%)	23 (17.3%)	13 (11.3%)	10 (55.6%)	
NEWS median (IQI)	4 (5)	3 (4)	8.5 (4.5)	<0.001
CURB-65 median (IQI)	1 (2)	1 (2)	3 (1.75)	<0.001

**Table 3:** Accuracy of 4C Mortality Score, NEWS, and CURB-65.

Score	AUC	*p*-Value	95% CI	
			Lower	Upper
4C Mortality score	0.818	<0.001	0.718	0.919
NEWS	0.861	<0.001	0.784	0.939
CURB-65	0.801	<0.001	0.681	0.922

**Table 4:** Diagnostic accuracy measures for mortality prediction at cut-offs of the 4C Mortality Score, NEWS, and CURB-65.

Cut-Off	Sensitivity (%)	Specificity (%)
4C Mortality Score		
>3	100%	24%
>8	94%	53%
>11	72%	67%
>14	55%	88%
NEWS		
>3	100%	51%
CURB-65		
>2	55%	92%

**Table 5:** Univariate logistic regression analysis of mortality risk scores.

Score	Odds Ratio	*p*-Value	95% CI	
			Upper	Lower
4C Mortality Score	1.33	<0.001	1.15	1.55
NEWS	1.56	<0.001	1.28	1.91
CURB-65	3.52	<0.001	1.95	6.38

## Data Availability

The data sets used and/or analyzed during the present study are available from the corresponding author on reasonable request.

## Appendix A

**Table A1:** List of abbreviations. The abbreviations including in the text are reported alphabetically.

Abbreviation	Full Form	Definition
ARDS	Acute respiratory distress syndrome	A life-threatening lung injury that allows fluid to leak into the lungs.
AUC	Area under the ROC curve	A performance measurement for the classification problems at various threshold settings.
CAP	Community-acquired pneumonia	An acute infection of the pulmonary parenchyma in someone who has not recently had close contact with the health care system.
CKD	Chronic kidney disease	Kidney damage or glomerular filtration rate (GFR) < 60 mL/min/1.73 m^2^ for 3 months or more, irrespective of cause.
COPD	Chronic obstructive pulmonary disease	A type of lung disease marked by permanent damage to tissues in the lungs, making it hard to breathe.
COVID-19	Coronavirus disease 2019	A respiratory disease caused by SARS-CoV-2, a coronavirus discovered in 2019.
ICU	Intensive care unit	The part of a hospital that provides intensive care.
IQI	Interquartile range	A measure of statistical dispersion.
.NEWS	National Early Warning Score	A tool developed by the Royal College of Physicians which improves the detection and response to clinical deterioration in adult patients.
ROC curve	Receiver operating characteristic curve	A graphical plot that illustrates the diagnostic ability of a binary classifier system as its discrimination threshold is varied.
WHO	World Health Organization	A specialized agency of the United Nations responsible for international public health.
